# SATURN: assessing the feasibility of utilising existing registries for real-world evidence data collection to meet patients, regulatory, health technology assessment and payer requirements

**DOI:** 10.1186/s13023-024-03341-4

**Published:** 2024-09-12

**Authors:** L. Sangiorgi, M. Boarini, M. Mordenti, V. Wang, I. Westerheim, R. T. Skarberg, M. Cavaller-Bellaubi, James Clancy, R. Pinedo-Villanueva, E. J. V. Lente, M. Marchetti

**Affiliations:** 1 Department of Rare Skeletal Disorders, IRCCS Istituto Ortopedico Rizzoli, Bologna, Italy; 2 UBC Late Stage Ltd, London, UK; 3 OIFE (Osteogenesis Imperfecta Federation Europe), Mechelen, Belgium; 4 European Reference Network On Rare Bone Diseases, Coordinating Centre, Bologna, Italy; 5 EURORDIS-Rare Diseases Europe, Patient Engagement and Therapeutic Development Director, Paris, France; 6 Mereo BioPharma Group Plc, London, UK; 7 Nuffield Department of Orthopaedics, Rheumatology and Musculoskeletal Sciences, University of Oxford, Oxford, UK; 8 Medicine Evaluation Committee, Brussels, Belgium; 9 Co-Chair European Member State Coordination Group On Health Technology Assessment, Italian Medicines Agency, Milan, Italy

**Keywords:** Feasibility assessment, Existing registries, REQueST, Gap analysis, Compliance check

## Abstract

**Background:**

SATURN (**S**ystematic **A**ccumulation of **T**reatment practices and **U**tilisation, **R**eal world evidence, and **N**atural history data) for the rare condition osteogenesis imperfecta (OI) has the objective to create a common core dataset by utilising existing, well-established data sources to meet the needs of the various stakeholders (physicians, registry/dataset owners, patients and patient associations, OI community leaders, European [EU] policymakers, regulators, health technology assessments [HTA]s, and healthcare systems including payers). This paper describes the steps taken to assess the feasibility of one existing OI registry (i.e., the Registry of OI [ROI]) as a candidate for SATURN. The same methodology will be applied to other existing OI registries in the future and this same concept could be utilised for other rare disease registries.

**Methods:**

The approach to assessing the feasibility of the ROI registry consisted of three steps: (1) an assessment of the registry characteristics using the Registry Evaluation and Quality Standards Tool (REQueST); (2) a gap analysis comparing SATURN required Core Variables to those being captured in the registry’s Case Report Form (CRF); and (3) a compliance check on the data exchange process following the Title 21 of Code of Federal Regulations (CFR) Part 11/EudraLex Annex 11 Compliance Checklist. The first registry that SATURN has assessed is the ROI database at the Istituto Ortopedico Rizzoli (IOR) in Italy.

**Results:**

The results from the ROI REQueST have demonstrated satisfactory complete responses in terms of methodology, essential standards, interpretability, and interoperability—readiness for data linkage, data sources, and ethics to meet the needs of data customers. However, the ROI data is from a tertiary referral centre which may limit the ability to understand the full patient journey. The gap analysis has revealed that an exact or logical match between SATURN requested variables and the ROI current variables exists for the following items: patient characteristics, treatment of OI (medical and surgical) and treatment of pain (with the exception of frequency of treatment and reasons for discontinuation), fracture history and bone density. However, data on safety was missing. The compliance check has implied that the ROI implemented appropriate controls for the web-based platform (i.e., Genotype–phenotype Data Integration Platform [GeDI]) that is involved in processing the electronic patient data, and GeDI is a validated/compliant application that follows relevant 21 CFR Part 11/EudraLex Annex 11 regulations.

**Conclusions:**

This robust feasibility process highlights potential limitations and opportunities to develop and to refine the collaboration with the ROI as the SATURN programme moves forward. It also ensures that the existing datasets in the rare condition OI are being maximised to respond to the needs of patients, data customers and decision-makers. This feasibility process has allowed SATURN to build a compliant methodology that aligns with the requirements from the European Medicines Agency (EMA) and HTAs. More data variables will continue to be developed and refined along the way with more registries participating in SATURN. As a result, SATURN will become a meaningful and truly collaborative core dataset, which will also contribute to advancing understanding of OI diagnosis, treatment, and care.

## Background

Treatments for rare diseases (RD)s face challenges in obtaining both regulatory approval and positive pricing and reimbursement decisions due to lack of documentation, smaller patient populations, the requirement of specific patient characteristics, uncertain or varying treatment guidelines (when available) and smaller markets for medicinal products.

Real-world data (RWD) is defined by the U.S. Food and Drug Administration (FDA) as “*data relating to patient health status and/or the delivery of health care routinely collected from a variety of sources including data derived from electronic health records, medical claims data, data from product or disease registries, and data gathered from other sources (such as digital health technologies) that can inform on health status*”; and real-world evidence (RWE) as “*clinical evidence about the usage and potential benefits or risks of a medical product derived from analysis of RWD*”  [7]. Whilst RWD should be a potential source of data to help address the challenges facing treatments for RDs, there is a limited amount of RWD available for RDs due to a long and difficult journey until an accurate diagnosis, lack of relevant diagnostic standards, limited knowledge by primary care and some specialists about the diseases, variance in treatment pathways, and the complicated patient journey.

The EMA has set out its vision that by 2025 the use of RWE will be a key and established contributor alongside data from other sources (including clinical trials) for a wide range of regulatory decision-making. This vision goes beyond the well-established uses in safety monitoring and epidemiology studies. It reflects a wider move by the EMA to leverage the value of RWE through its Big Data Steering Group initiative and workstreams – including ensuring data quality, discoverability, and governance, which are key elements of the existing EMA registry-based studies guidance [1]. The EMA has also developed the Data Analysis and Real World Interrogation Network (DARWIN) Project for hosting a catalogue of observational data sources [2].

If RWD is to be utilised to this magnitude, data must be of a sufficiently robust standard to be meaningful. The EUnetHTA has developed a tool, REQueST, which sets out standards that are universal and essential for good practice and data quality and which are relevant for different types of registries [3]. REQueST is designed to be broken into three distinct stages [4]:

- Methodology: type of registry, previous-use and publications, geography and organisational setting, duration, size, inclusion/exclusion criteria, follow-up, confounders;
- Essential Standards: aims and methodology, governance, informed consent, data dictionary, minimum dataset, standard definitions, terminology and specification, data collection, quality assurance, data cleaning, missing data, financing, protect, security and safeguards;
- Additional Requirements: interoperability and readiness for data linkage, data sources and ethics.

It is clear from these regulatory initiatives that there will be increasing value placed upon RWD in the future. Such data, when robust and compliant with required standards, will have a valuable role in the RD arena in providing much needed information to support treatments for patients with RD.

SATURN has the objective to create a common core dataset by utilising existing, well-established real world data sources, to meet the needs of the various stakeholders (physicians, registry/dataset owners, patients and patient associations, OI community leaders, EU policymakers, regulators, HTAs, and healthcare systems including payers).

SATURN will align with the EMA guidance that ensures the availability of the data, that feasibility assessments are undertaken, and that quality control measures and procedures are executed to support the quality of data outputs. A framework will be established to oversee and manage SATURN – including a Governance Plan, a Data Management Plan (DMP) that is in alignment with findable, accessible, interoperable, and reusable (FAIR) principles [8, 11], and a Quality Management Plan and Research Protocol.

The concept of SATURN has been described previously [10]. Here we describe the methodology for its development in terms of governance, data management, quality, and compliance to ensure that the outputs from SATURN will be relevant and reliable for decision-makers at different stages of a medicines’ evaluation and availability pathways. We specifically describe the methodology and results of a feasibility assessment undertaken of ROI as part of SATURN. The ROI, established at the IOR, one of the leading institutions for OI in Italy, is the first registry being assessed under SATURN umbrella. It consists of a well-established dataset [9] gathering records from approximately 1300 patients with OI, using a robust and validated cloud-based electronic platform. All patients treated at Department of Rare Skeletal Disorders of IOR, who match the inclusion criteria of the ROI protocol, have been enrolled in this registry.

The outcome of this assessment will allow better understanding of patient characteristics, variables captured, population size, data quality, data governance, and data sharing policies. This exercise will then be repeated with other datasets/registries for OI. A full feasibility report will be produced for each registry. This approach will ensure a consistent and complete approach to each registry and secure that the datasets being used in SATURN to generate aggregated data outputs are of the required quality and consistency.

## Methods

**Fig. 1:**
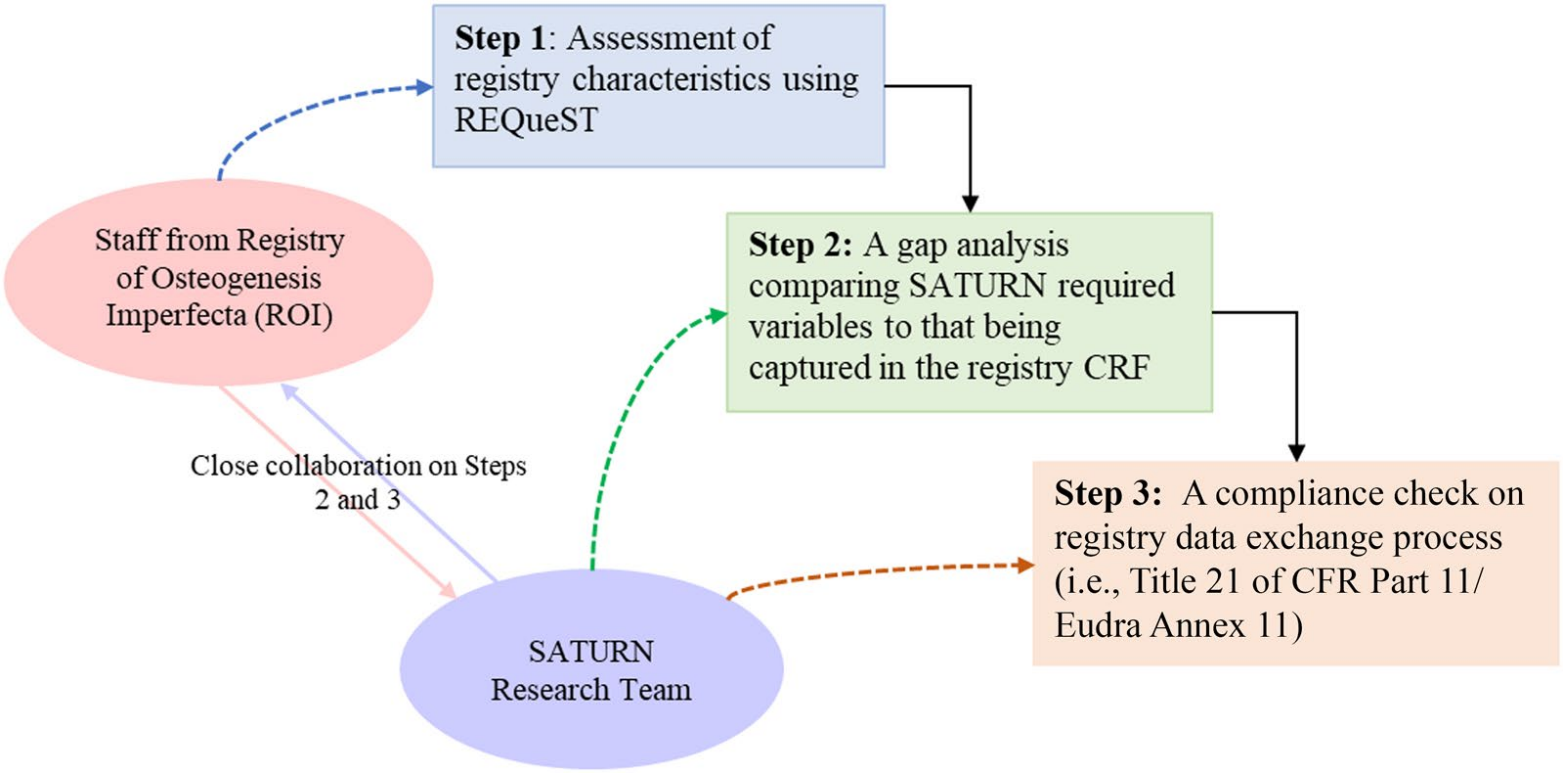
SATURN—a Stepwise Approach to Assessing the Feasibility of the ROI. CFR, Code of Federal Regulations; CRF, case report form; REQueST, Registry Evaluation of Quality Standards Tool; ROI, Registry of Osteogenesis Imperfecta; SATURN, Systematic Accumulation of Treatment practices and Utilisation, Real world evidence, and Natural history data

The approach to assessing the feasibility of the ROI registry consisted of three steps: 1) an assessment of the registry characteristics using REQueST; 2) a gap analysis comparing SATURN required Core Variables to those being captured in the registry’s CRF; and 3) a compliance check on the data exchange process following the Title 21 of CFR Part 11 [6]/EudraLex Annex 11 [5] (Fig. 1).

*Step 1* The assessment using REQueST consists of three stages: Methodological Information, Essential Standards and Additional Requirements. The tool was sent by the SATURN Research Team (SRT)^1^ to the IOR for their completion. Once completed, it was returned to the SRT for review. A dialogue subsequently took place between the two parties to resolve any outstanding issues. The outcome was documented in REQueST. For the Methodological Information and Additional Requirements stages, the SRT made a final assessment as to whether the responses would meet HTA’s/regulators requirements (Table 1) and for the Essential Standards section, the SRT made a final assessment as to whether the minimum standard was met. In all stages, the SRT added additional comments in relation to SATURN as applicable (Table 2).

**Table 1:** Completion of Methodological Information and Additional Requirements of REQueST

Area	Item and format required	Column to be completed by the registry owner—with hyperlinks to relevant online documents where possible	Does the information meet the HTA agency/regulator's needs?	Comment in relation to SATURN requirements
Determined by REQueST	Determined by REQueST	Completed by ROI Subsequent documented dialogue between the ROI and SRT	Completed by SRT	Completed by SRT

HTA, Health Technology Assessment; REQueST, Registry Evaluation of Quality Standards Tool; ROI, Registry of Osteogenesis Imperfecta; SATURN, Systematic Accumulation of Treatment practices and Utilisation, Real world evidence, and Natural history data; SRT, SATURN Research Team

**Table 2:** Completion of Essential Standards of REQueST

Area	Minimum Standard	Assessment Criteria	Item and format required	Column to be completed by the registry owner—with hyperlinks to relevant online documents where possible	Is the Minimum Standard met?	Comment in relation to SATURN requirements
Determined by REQueST	Determined by REQueST	Determined by REQueST	Determined by REQueST	Completed by ROI Subsequent documented dialogue between the ROI and SRT	Completed by SRT	Completed by SRT

HTA, Health Technology Assessment; REQueST, Registry Evaluation of Quality Standards Tool; ROI, Registry of Osteogenesis Imperfecta; SATURN, Systematic Accumulation of Treatment practices and Utilisation, Real world evidence, and Natural history data; SRT, SATURN Research Team

*Step 2* The draft Core Variables List was initially based on clinical trial endpoints for a product in development for OI. In an RWE setting, it was assessed by the ROI team and then adjusted following their feedback.

In this feasibility assessment, the draft Core Variables List was compared to the registry variables contained in the data dictionary/CRF utilised by the ROI. The gap analysis, undertaken by a data management specialist of the SRT, involved a manual comparison to identify the similarities and differences in registry variables contained in the CRF vs. the draft Core Variable List. In this analysis, each variable in the draft Core Variables List was categorised as either “Exact Match”, “Logical Match”, or “Omitted” to reflect the status in the ROI.

- Exact Match: registry contains the same variable and variable responses as the draft Core Variables List.
- Logical Match: registry contains a similar variable and variable responses as the draft Core Variables List for analysis. Special scenarios include:
  - Exact variable/variable response not collected in the registry; however, required variables can be derived using other information collected. For example, “patient age” not collected in registry; however, as “date of birth” and “date of consent” were captured, these two variables can be used to derive patients’ age.
  - Minor differences in variable format were allowed. For example, “surgery type” was not captured in registry as free text, however, registry captures “surgery type” using a pre-defined updatable list of surgeries in a drop-down menu.
- Omitted: registry does not contain the variable within the draft Core Variables List.

Once completed, the results of the gap analysis were reviewed by the SRT and then by the ROI. In addition, the ROI was required to confirm the list of omitted variables. The final decision on the degree of variable matching was made by the full team and alignment was reached after consolidating all feedback from the SRT and the ROI.

*Step 3* A compliance check on the data exchange process following the Title 21 of CFR Part 11/EudraLex Annex 11 Compliance Checklist was performed under the collaboration between both the ROI staff and SRT.

The checklist was used to determine if the ROI implemented controls, including audits, system validation, audit trials, electronic signatures, and documentation for the web-based platform GeDI that was involved in processing the electronic patient data.

More specifically, the project manager of the SRT assessed the ROI data source characteristics and answered the following questions:

- Does the project generate information and/or data that is used in any decision-making process during the Research Development, or Clinical process?
- Will the project be used to support regulatory/HTAs/payer submissions?
- Does the project process, transfer or store Good Clinical Practice (GCP)-related information in electronic format that is required by regulatory agencies?
- Could the project have an impact on an already qualified/validated environment that supports regulatory data?
- Does GCP apply to the system?

If any were answered “Yes”, a compliance check on electronic data exchange process was required. The ROI research staff then received the checklist from SRT and conducted the compliance check by assessing all items on the 21 CFR Part 11/EudraLex Annex 11 Compliance Checklist.

## Results

### Step 1: REQueST

Responses to REQueST are provided in Table 3. All responses in the Methodological Information and Additional Requirements were considered by the SRT to meet HTA agency/payers/regulator's needs and all responses in the Essential Standards were considered to have met the Minimum Standards.

**Table 3:** The ROI Characteristics using REQueST

Item number	Area	The ROI Assessment Response
Methodological Information		
1	Type of registry	The ROI is a disease registry containing observational data of patients suspected and/or affected by OI
2	Use for registry-based studies and previous publications	Detailed information on diagnosis, clinical symptoms, surgeries, laboratory analyses, genetic analysis and treatments, side effects, patient-reported outcomes (PROs), and long-term outcomes are collected from first access onward. Data entry continues throughout the year by healthcare professionals and registry team members using several data sources (CRF paper-based form, medical report, etc.) then captured according to the ROI data dictionary on a web-based platform, GeDI Previous publications: - Maioli M, Gnoli M, Boarini M, et al. Genotype–phenotype correlation study in 364 osteogenesis imperfecta Italian patients. *European Journal of Human Genetics*. 2019;27:1090–1100 - Mordenti M, Boarini M, D’Alessandro F, et al. Remodeling an existing rare disease registry to be used in regulatory context: lessons learned and recommendations. *Frontiers in Pharmacology.* 2022 (13):966081
3	Geographical and organisational setting	National coverage in Italy. Data providers include clinical units (within hospitals, outpatient clinics, university hospitals), laboratories/central services (pathological services, genetic); centres of expertise (public and private)
4	Duration	25 years and ongoing
5	Size	Approximately 1300 patients with OI (by June 2024)
6	Inclusion and exclusion criteria	Inclusion criterion: - Patients with OI, including prenatal and foetal diagnosis of OI Exclusion criterion: - Any condition unrelated to OI Study Details | Registry of Osteogenesis Imperfecta | ClinicalTrials.gov
7	Follow-up	On average every 12–18 months The follow-up periods are dependent on disease requirements and consistent with clinical manifestations and/or surgical treatments. The follow-up period may vary greatly among individual patients (e.g., patient visit on an as-needed basis vs. patients with regular routine check-ups)
8	Confounders	The ROI collects patient demographics, comorbidities, and concomitant therapies. At present, these data are not disclosed to the appropriate Market Authorisation Holder
Essential Standards		
9	Registry aims and methodology	Aims to establish a disease registry to understand the natural history of the OI. Clinical, functional, rehabilitation, radiological surgical, treatment, genetic, genealogical and quality of life data and biological samples were collected from patients’ medical records
10	Governance	The governance of the ROI involves a group of clinicians, technicians, researchers, as well as data manager, data curator, quality manager, and registry manager
11	Informed consent	Informed consent is mandatory to patient enrolment. Data sharing agreement was also provided
12	Data dictionary	Data dictionary provided
13	Minimum data set	The ROI is developed based on the Common Data Elements that are recommended by European standards for data collection (https://eu-rd-latform.jrc.ec.europa.eu/set-of-common-data-elements_en) Data on diagnosis, disease history and care pathway, information for research purposes and disability etc. were collected
14	Standard definitions, terminology and specifications	The ROI is consistent with national and international data standards. Terminologies and ontologies are listed in CRF Data Dictionary
15	Data collection	Routine clinical data were collected in accordance with local protocol and entered in the patients’ hospital notes. Data were transferred to the database
16	Quality assurance	Data were double checked by two separate users at first entering. Additionally, sample checks are performed by registry staff every month or on request (i.e., platform release, data extractions, etc.)
17	Data cleaning	Registry staff re-check all the information collected at every patient’s follow-up. In addition, the system is implemented to highlight the duplicates, the lack of mandatory data and data inconsistency
18	Missing data	To minimise the amount of missing data, registry staff re-checks all the information collected at every patient’s follow-up. In addition, patients are asked to share integrative information on their disease (medical reports, imaging, etc.)
19	Financing	All the costs related to the ROI maintenance, platform implementation, training, personnel, etc. are institutionally funded
20	Protection, security and safeguards	(1) All patient data are pseudonymised; (2) The database is fully compliant with Data Protection legislation (GDPR and Italian national regulation)
Additional Requirements		
21	Interoperability and readiness for data linkage	Specific agreement is drafted for access, sharing and related fees in collaboration with the legal and administrative institutional offices for research purpose
22	Data sources	Patient medical records
23	Ethics	The ROI was approved by Institutional Review Board of Istituto Ortopedico Rizzoli in 2013

CRF, case report form; GDPR, General Data Protection Regulation; GeDI, Genotype–phenotype Data Integration Platform; OI, osteogenesis imperfecta; PRO, patient-reported outcomes; ROI, Registry of Osteogenesis Imperfecta

### Step 2: Gap analysis

Table 4 sets out the high-level results of the gap analysis and shows for which of the draft SATURN Variables there was either an Exact Match, Logical Match in the ROI CRF or whether the variable was omitted.

**Table 4:** Gap analysis of the ROI dataset compared to required SATURN variables

Category	Draft SATURN Variables	The ROI Match
Registry Consent	Consent data	Exact Match
Patient Characteristics	Age	Logical Match
	Sex	Logical Match
	OI diagnosis date	Logical Match
	OI type	Logical Match
	Genotype	Logical Match
	OI—Symptoms and conditions	Logical Match
Treatment for OI	Drug name	Logical Match
	Start date	Exact Match
	End date	Exact Match
	Frequency of treatment	Omitted
	Reason for discontinuation	Omitted
	Surgery	Logical Match
	Date of surgery	Exact Match
Treatment for Pain	Drug name	Logical Match
	Start date	Logical Match
	End date	Logical Match
	Frequency of treatment	Omitted
	Reason for pain treatment	Omitted
	Reason for discontinuation	Omitted
Fracture History Collection	Time of first fracture	Logical Match
	Approximate total number of fractures experienced in lifetime	Exact Match
	Fracture locations	Logical Match
	Number of fractures in this location	Logical Match
	Date of last fracture in this location	Logical Match
Fracture Collection (prospectively)	Fraction Location	Logical Match
	Date of fracture	Logical Match
	Fracture Treatment	Logical Match
	Impact of fracture / QoL impact	Logical Match
Assessments	DXA / X Ray date	Omitted
	DXA / X Ray outcome	Logical Match
Safety	ADRs Term	Omitted
	ADRs start date	Omitted
	ADRs end data	Omitted
	ADR Ongoing?	Omitted
	TCAE Grade	Omitted
	Relationship with drug	Omitted
	Specify drug	Omitted
	Action taken with drug	Omitted
	Outcome	Omitted
	Serious ADR	Omitted
Quality of Life	Patient reported	Logical Match
	Parent/care giver	Logical Match
Health Resource Utilisation	Has the patient ever been hospitalised for their OI	Logical Match
	Has the patient been hospitalised for their OI	Logical Match
	How many nights in hospital have been spent as part of the hospitalisation	Omitted
	How many nights spent in ITU as part of the hospitalisation	Omitted
	Has the patient had to visit A&E / Emergency Room	Logical Match
	Reason for visit to A&E / Emergency Room	Logical Match
	Has the patient required any other healthcare providers	Logical Match
	Which healthcare provider	Logical Match
	How many times have they visited the healthcare provider	Logical Match

ADR, adverse drug reaction; A&E, accident and emergency; DXA, dual-energy x-ray absorptiometry; ITU, intensive therapy unit; QoL, quality of life; OI, Osteogenesis Imperfecta; ROI, Registry of Osteogenesis Imperfecta; SATURN, Systematic Accumulation of Treatment practices and Utilisation, Real world evidence, and Natural history data; TCAE, Terminology Criteria for Adverse Events

Whilst an Exact Match and Omitted are clearly defined measures, logical matches are subject to individual opinion. The rationale for categorizing a variable as a logical match is set out in in Table 5.

**Table 5:** Logical Matched Variables

Category	Draft Core Variables requested for SATURN	Rationale for logical match
Patient Characteristics	Age (YY)	Age can be derived based on DOB and date of consent
Patient Characteristics	Sex (M or F)	The logically matched the ROI variable is “Patient-gender at birth” with responses “M/F/Unknown”
Patient Characteristics	OI diagnosis date (mm/yyyy)	Minor difference in response format The logically matched the ROI variable is “Patient characteristics – diagnosis date (dd/mm/yyyy)
Patient Characteristics	OI type (type I, III, IV, etc.)	Minor difference in response format The logically matched the ROI variable is Patient Characteristics- Diagnosis Type (Drop-down single-choice list)
Patient Characteristics	Genotype (free text)	Minor difference in response format The logically matched the ROI variable is “Genetic Analysis: Gene”
Patient Characteristics	OI—Symptoms and conditions	The logically matched the ROI variable is “Patient Characteristics- Musculoskeletal Sys, Cardio & Lymph Sys, Dysmorphism, Sensory Organs, Nervous Sys, Other Apparatuses, Functional Assessment, Sign/Symptom Manifestation, Deformity/Limitation”
Treatment for OI	Drug name (free text, to include all treatment, bisphosphonates/bone anabolic agents)	Minor difference in collected variable name The logically matched the ROI variables is “Treatment: Pharmacotherapy-Drug Name-Comm Name-Free text”
Treatment for OI	Surgery (free text, to include Ostectomy/rodding surgery/revision surgery/surgical fracture repair/Removal of surgery fracture repair/spinal fusion/occipito-cervical bracing/hearing loss related corrective surgery)	Minor difference in response format The logically matched the ROI variable is “Tab = Surgery: Surgery Type- Several Ontology Code (Drop-down single-choice list)”
Treatment for Pain	Drug name (free text)	The logically matched the ROI variable is “Tab = Treatment: Pharmacotherapy – Drug Name – Commercial name” in combination with “Tab = Treatment: General”
Treatment for Pain	Start date	The logically matched the ROI variable is “Tab = Treatment: Pharmacotherapy – Start Date”
Treatment for Pain	End date	The logically matched the ROI variable is “Tab = Treatment: Pharmacotherapy – End Date”
Fracture History Collection	Time of first fracture (dd/mm/yyyy)	The logically matched the ROI variable is “Tab = Clinical Event; Question = Clinical Event Date”
Fracture History Collection	Fracture locations (free text or drop-down list)	The logically matched the ROI variable is “Tab = Clinical Event: Patient Char- Musculoskeletal Sys”
Fracture History Collection	Number of fractures in this location	The logically matched the ROI variable is “Tab = Clinical Event: Patient Char—Sign/Symptom Manifestation—Fracture Number (Number of fractures in specific site)”
Fracture History Collection	Date of last fracture in this location (dd/mm/yyyy)	Minor difference in collected variable name. The logically matched the ROI variable is “age at last fracture in this location”
Fracture Collection (prospectively)	Fracture Location (free text)	The logically matched the ROI variable is “Tab = Clinical Event: Patient Char – Sign/Symptom Manifestation – Site/Side/Localisation (MeSH code)”
Fracture Collection (prospectively)	Date of fracture (dd/mm/yyyy)	Minor difference in collected variable name. The logically matched the ROI variable is “age at fracture”
Fracture Collection (prospectively)	Fracture Treatment (surgery/plaster/bandage)	The logically matched the ROI variable is “Tab = Surgery: Treatment” or “Tab = Treatment”
Fracture Collection (prospectively)	Impact of fracture /QoL of fracture	The logically matched the ROI variable is “Tab = PROs: QOL- EQ-5D (Adult or Children Version)—Mobility, Self-Care, Daily Act., Pain/Discomfort, Anxiety/Depression,—Drop down single choice list, VAS (Global health status scale 0–100)”
Assessments	DXA/X Ray outcome	The logically matched the ROI variable is “Tab = Clinical Event: Pt. Char- Musculoskeletal Sys- BMD, BMD Z-score, BMD T-score—DXA Report- Number”
Quality of Life	Patient reported	The logically matched the ROI variable is “Tab = PROs: QOL- EQ-5D (Adult or Children Version)—Mobility, Self-Care, Daily Act., Pain/Discomfort, Anxiety/Depression,—Drop down single choice list, VAS (Global health status scale 0–100) Didn't specify Patient Reported.”
Quality of Life	Parent/care giver?	The logically matched the ROI variable is “Tab = PROs: QOL- EQ-5D (Adult or Children Version)—Mobility, Self-Care, Daily Act., Pain/Discomfort, Anxiety/Depression,—Drop down single choice list, VAS (Global health status scale 0–100) Didn't specify Patient Reported.”
Health Resource Utilisation	Has the patient ever been hospitalised for their OI	The ROI confirmed that specific HRU information not collected, but some information can be obtained from the Signs & symptoms/ treatment tabs
Health Resource Utilisation	Has the patient been hospitalised for their OI	The ROI confirmed that specific HRU information not collected, but some information can be obtained from the Signs & symptoms/ treatment tabs
Health Resource Utilisation	Has the patient had to visit A&E /Emergency Room	The ROI confirmed that specific HRU information not collected, but some information can be obtained from the Signs & symptoms/ treatment tabs
Health Resource Utilisation	Reason for visit to A&E / Emergency Room	The ROI confirmed that specific HRU information not collected, but some information can be obtained from the Signs & symptoms/ treatment tabs
Health Resource Utilisation	Has the patient required any other healthcare providers	The ROI confirmed that specific HRU information not collected, but some information can be obtained from the Signs & symptoms/ treatment tabs
Health Resource Utilisation	Which healthcare provider	The ROI confirmed that specific HRU information not collected, but some information can be obtained from the Signs & symptoms/ treatment tabs
Health Resource Utilisation	How many times have they visited the healthcare provider	The ROI confirmed that specific HRU information not collected, but some information can be obtained from the Signs & symptoms/ treatment tabs

A&E, accident and emergency; BMD, bone mineral density; DOB, date of birth; DXA, dual-energy x-ray absorptiometry; F, female; HRU, healthcare resource utilisation; M, male; MeSH, Medical Subject Headings; OI, Osteogenesis Imperfecta; PRO, patient-reported outcome; QOL, quality of life; ROI, Registry of Osteogenesis Imperfecta; VAS, visual analogue scale

### Step 3: A compliance check on the data exchange process

Per ROI’s statements, the ROI confirmed that that their system (i.e., GeDI) that captures the patient data complies with relevant 21 CFR Part 11/EudraLex Annex 11 Compliance Checklist. Electronic signatures are out of scope as it is not a part of the GeDI platform functionality. Detailed results of the compliance check on the ROI data exchange process are provided in Appendix A. Compliance Check on the ROI Electronic Data Exchange.

## Discussions

The feasibility assessment has been based upon a review of REQueST, the gap analysis comparing the draft data variables identified by SATURN and those collected in the CRF of the ROI, and a compliance check. The results from the ROI REQueST have demonstrated satisfactory complete responses in terms of methodology, essential standards, interpretability, and interoperability—readiness for data linkage, data sources, and ethics to meet the needs of data customers.

However, a few points have been highlighted during the feasibility assessment which may result in some limitations. Firstly, it is stated in the responses that the registry has national coverage, but it is later noted that the majority of OI patients are from one institution. This is because the ROI is a referral centre for OI management. Thus, although patients may reside throughout Italy, they are seen when necessary at this tertiary centre. The frequency of visits can vary depending on the need to be seen at this tertiary centre. This can vary from several times a year to once every 10 years and differs from the primary or secondary care setting where both children and adults would have regular follow up and regular data collection.

The implications of this could be that (1) the data will only reflect the visits to the tertiary centre and not the full patient treatment journey; (2) visits may only occur as needed and not on a regular basis; and (3) regional variations in treatment may not be captured.

Secondly, in REQueST it is stated that comorbidities and concomitant therapies are collected but not disclosed to the appropriate Marketing Authorisation Holder. This comment needs further clarification as this could limit the objectives of a specific study in the future.

A draft Core Variables List was developed initially from OI clinical trials’ endpoints. In this feasibility study, it was assessed by the ROI team and then revised following feedback based on their real-world clinical practice experiences. As the SATURN programme moves forward, ongoing validations of this draft Core Variables List will be undertaken with future collaborations and assessment by Key Opinion Leaders (KOLs). The final Core Variables List aims to include all data-points which would be required to meet the needs of physicians, registry owners, patients, OI community leaders, EU policymakers, regulators, HTAs and healthcare systems including payers.

In this feasibility assessment, the gap analysis has revealed that an exact or logical match between SATURN requested variables and the ROI current variables exists for the following items: patient characteristics, treatment of OI (medical and surgical) and treatment of pain (with the exception of frequency of treatment and reasons for discontinuation), fracture history and bone density. However, there is a significant gap in relation to the collection of safety data, as no safety data is captured in the ROI at present. This is understandable as the ROI has the objective of observing the natural history of OI rather than assessing safety of specific treatments. Potential inclusion of additional fields in the future, such as safety data, would need to be agreed with ROI.

Whether or not the ROI can be used as a complete data source for SATURN will depend upon the final objectives of any study protocol and whether the ROI is amenable to integrating additional data variables into their established data collection infrastructure to achieve customised data collection.

Based upon the feasibility results, a study to understand the natural history and treatment pathway of patients with OI appears to be feasible because variables of OI type, genotype, symptoms, and treatment are included. A limitation is that frequency of pharmaceutical treatments and reasons for discontinuation are currently not included. However, it is anticipated that these variables would not be critical for such a study.

A study to assess health-related quality of life (HRQoL) may also be feasible, but this would be limited to EQ-5D, and pain score measured by age and site-specific visual analogue scale (VAS) which are currently already collected within the ROI.

The ROI does not collect healthcare resource utilisation (HRU) data specifically although has stated that some information can be obtained from the signs & symptoms/ treatment fields. Further investigation would be needed to determine if this information would be sufficient to address any research questions concerning HRU.

Should there be a regulatory request for the sponsor of SATURN to undertake a post-authorisation safety study (PASS), the ROI, with its current data collection parameters, would need to add adverse event (AE) variables to the data collected and this would require the agreement of the data owner. However, it may be possible to undertake a post authorisation effectiveness study (PAES) because endpoints such as fracture history and bone density are included. If the ROI data collection is enhanced in the future with additional safety information, then this data source could be valuable for PASS studies.

Finally, it should be noted that the feasibility assessment does not give any indication of the completeness of the data collected. Data will be entered into the ROI registry as a reflection of the normal clinical practice of the treating physician and hence there is always the possibility of missing data if a particular parameter was not clinically assessed or recorded. The extent of missing data cannot be predicted from the feasibility assessment. SATURN plans to obtain data from the ROI registry in order to assess the completeness of the data to answer future research questions.

In parallel with ongoing dialogue with the “data customers”, i.e. regulators, HTA bodies or national payers/budget-holders, SATURN aims to be able to provide aggregated data outputs to answer decision-makers’ questions in a robust and high-quality manner.

At this moment, these results can only reflect the ROI analysis. However, further databases will be added in the future, which will make SATURN more representative across Europe.

## Conclusions

This robust feasibility process highlights potential limitations and opportunities to develop and to refine the collaboration with the ROI as the SATURN programme moves forward. It also ensures that the existing datasets in the rare condition OI are being maximised to respond to the needs of patients, data customers and decision-makers.

REQueST standardises the approach to assess each registry. It allows for a clear understanding of the registry and data collection process, and an assessment of each essential standard to ensure that the quality and breadth of data will meet data customers’ requirements.

The variable gap analysis between the ROI variables and SATURN dataset produces a clear overview of the availability of data, the data variables collected, and any gaps currently identified which could be addressed as the collaboration continues and as SATURN develops to meet the broader needs of the data customers.

The compliance check ensures that the ROI implements appropriate controls for the web-based platform that is involved in processing the electronic patient data and follows relevant 21 CFR Part 11/EudraLex Annex 11 regulations.

Completeness of data was not assessed in this feasibility assessment, but SATURN plans to analyse an output of the data in the future.

This feasibility assessment has allowed SATURN to begin to build a compliant methodology that aligns with the requirements from the EMA and HTAs. More data variables will continue to be developed and refined along the way with more registries participating in SATURN and with the data customers (e.g., patients, regulators, HTAs, healthcare systems including payers). As a result, SATURN will become a meaningful and truly collaborative core dataset, which will also contribute to advancing understanding of OI diagnosis, treatment and care.

## Data Availability

Not applicable.

## Appendix A: Compliance Check on the ROI Electronic Data Exchange Process

**Table:**

System Name & Version	Genotype–phenotype Data Integration Platform (GeDI) v.3.4.2
Date of Assessment Initiation	24 October 2023
Date of Assessment Completion	24 January 2024
Brief Description of System Functionality	GeDI is a standardised and web-accessible platform that stores clinical, genetic, family history as well as quality of life data from patients. It is built in compliance with national and European privacy regulation and medical informatic standards
System Development	☒ Internally developed ☒ Vendor Purchased ☐ Off-the-shelf
System Classification	☐ Open ☒ Closed
Are electronic signatures used?	☐ Yes ☒ No, (Section II not required)

**Table:**

21CFR11/ EudraLex Annex 11 Ref	Item	Yes/No	Comment: Please list SOPs where appropriate
11.10 (a)	Has the system been validated to ensure accuracy and reliability is consistent with the intended performance?	☒ Yes ☐ No	
	(a) Are documented user/functional requirements available to support the validation testing?	☒ Yes ☐ No	
	(b) Is there documented evidence to support the validation effort?	☒ Yes ☐ No	
	Has the system been validated to discern altered or invalid records?	☒ Yes ☐ No	
	(a) Was a vendor audit performed?	☒ Yes ☐ No	
	(b) Was the system developed and tested according to a documented SDLC?	☒ Yes ☐ No	
	(c) Was User Acceptance testing performed in accordance with UBC procedures?	☐ Yes ☐ No	N/A
11.10 (b)	Can accurate and complete copies of records be generated in both electronic and human readable hard copy format?	☒ Yes ☐ No	
11.10 (c)	Are **all** records protected to ensure accurate and ready retrieval throughout the record retention period?	☒ Yes ☐ No	
	(a) Are system-specific documented procedures for system back up and restoration available?	☒ Yes ☐ No	
	(b) Were the backup/restoration procedures tested as part of the validation process?	☒ Yes ☐ No	
	(c) Can the audit trail be printed?	☐ Yes ☒ No	
11.10 (d)	Does this system limit access to authorised personnel?	☒ Yes ☐ No	
	(a) Define the non-biometric or biometric controls in place for system access	☒ Yes ☐ No	Non-biometric via log in (username&password)
	(b) Can user profiles be defined within the system to limit access to certain functionality?	☒ Yes ☐ No	
	(c) Are internal SOPs in place regarding user access administration (e.g. assigning, modifying, and deleting user accounts/profiles)? List applicable SOPs	☒ Yes ☐ No	IO01MR Inserimento dati GeDI rev03
	(d) Are SOPs in place to document user profiles within the system? List applicable SOPs	☒ Yes ☐ No	IO01MR Inserimento dati GeDI rev03
11.10 (e)	Does the system have secure, computer-generated time-stamped audit trails to independently record the date and time of operator entries and actions that create, modify, or delete electronic records?	☒ Yes ☐ No	
	(a) Are audit trails created incrementally, in chronological order, and in a manner that does not allow new audit trail information to overwrite existing data?	☒ Yes ☐ No	
	(b) Are personnel who create, modify, or delete electronic records able to modify the audit trails?	☐ Yes ☒ No	
	(c) Is the audit trail documentation retained for a period at least as long as that required for the subject electronic records?	☒ Yes ☐ No	
	(d) Is audit trail documentation available for agency review and copying (e.g. can it be printed in hard copy or copied in electronic format)?	☒ Yes ☐ No	It can be exported in.csv
	(e) Are date and times local to the activity being documented and include year, month day, hour, and minute or maintained in a central time zone which is documented? (Note: Calculation of the local time stamp in certain instances may be derived from a remote server located in a different time zone.)	☒ Yes ☐ No	
	(f) Are controls in place to ensure that the system’s date and time are correct?	☒ Yes ☐ No	An NTP server managed and monitored by the Rizzoli System Administrator is configured on all systems/devices connected in the Institutional network
	(g) Is the ability to change the date or time limited to authorised personnel?	☒ Yes ☐ No	The systemic tasks/activities are the sole responsibility of system administration technicians
	(h) Are SOPs in place for the reporting, correction, and documentation of date/time discrepancies noted?	☒ Yes ☐ No	A monitoring platform has been implemented for all systems/devices in the Institutional network, allowing the system technicians to intervene 24/7 for malfunction/failure
	(i) Are all changes to the record maintained incrementally such that the original and current values are retained?	☒ Yes ☐ No	
	(j) Is the name of the user, time of change, and reason for change documented in the audit trail?	☒ Yes ☐ No	
	(k) Is the name of the user recorded by the system based on the login identification codes?	☒ Yes ☐ No	
11.10 (f)	Are there operational system checks to enforce permitted sequencing of steps and events?	☒ Yes ☐ No	
	(a) Do the system functionality and workflow support UBC procedures?	☐ Yes ☐ No	N/A
	(b) Are warning messages programmed in the system to notify the user of invalid/skipped processes?	☒ Yes ☐ No	
11.10 (g)	Are authority checks used by the system to ensure that only authorised individuals can use the system, electronically sign a record, access the operation or computer system input or output device, alter a record, or perform the operation at hand?	☒ Yes ☐ No	
	(a) Are all users authenticated at log on?	☒ Yes ☐ No	
	(b) Does this “time-out” if not in use for a specified period of time?	☒ Yes ☐ No	After 60 mins the user is logged off
	(c) Is the name of the user present on-screen during system use?	☒ Yes ☐ No	
11.10 (h)	Are device (e.g. terminal) checks used to determine, as appropriate, the validity of the source of data input or operational instruction?	☐ Yes ☐ No	N/A. Data are collected from different sources (paper-based and/or other software). Nevertheless, data are manually input into the GeDI platform
	(a) Are routine checks being performed to determine if the source data is valid?	☐ Yes ☐ No	N/A
11.10 (i)	Is documentation of education, experience, and training (including training on use of the system and security) available for End Users?	☒ Yes ☐ No	
	(a) Has system training been documented for all current users **prior** to processing live data?	☒Yes ☐ No	
	(b) Are SOPs in place defining the training requirements for the system?	☐ Yes ☒ No	
	(c) Have all users been trained in accordance with the SOPS defined above?	☒ Yes ☐ No	IO01MR Inserimento dati GeDI rev03
	(d) Are CVs on file for all system users?	☒ Yes ☐ No	
	(e) Have all IT support staff been trained to adequately maintain the system?	☒ Yes ☐ No	
11.10 (j)	Are written policies to hold individuals accountable and responsible for actions initiated under their electronic signatures, in order to deter record and signature falsification?	☐ Yes ☒ No	
	(a) Is there evidence of training/agreement of all system users?	☒ Yes ☐ No	
	(b) Are periodic audits/reviews performed to monitor compliance with the above policy?	☒ Yes ☐ No	
11.10 (k)(1)	Are there adequate controls over documentation for system operation and maintenance with respect to distribution, access, and use?	☒ Yes ☐ No	
	(a) Is original documentation maintained in a secured area?	☒ Yes ☐ No	The original documents are both paper-based and electronically based. All those documents are in secured area
	(b) Is documentation provided to all users?	☒ Yes ☐ No	
11.10 (k)(2)	Are there adequate controls over system documentation used for revision and change control procedures that maintain an audit trail for documenting time-sequenced development and modification of systems documentation?	☒ Yes ☐ No	
	(a) Do formal procedures regarding change control of system documentation exist?	☐Yes ☒ No	
	(b) Is each version of software accompanied by new system documentation (e.g. release notes from vendor purchased systems)?	☒ Yes ☐ No	Ticketing system
	(c) Are all users notified of new documentation/ procedures?	☒ Yes ☐ No	
	(d) Is documentation for older versions maintained in a secured environment?	☒ Yes ☐ No	
11.30	Are document encryption and appropriate digital signature standards used to ensure record authenticity, integrity, and confidentiality?	☒ Yes ☐ No	Just the encryption
11.50 (a)	Do the signed electronic records contain information associated with the signing that clearly indicates:	☐Yes ☐ No	N/A
11.50 (a)(1)	• The printed name of the signer?	☐ Yes ☐ No	N/A
11.50 (a)(2)	• The date and time when the signature was executed?	☐ Yes ☐ No	N/A
11.50 (a)(3)	• The meaning (such as review, approval, responsibility, or authorship) associated with the signature?	☐ Yes ☐ No	N/A
	(a) Are documented procedures available which define how electronic signatures are executed and meaning of each signature? List applicable SOPs	☐ Yes ☐ No	N/A
	(b) Is the electronic signature subject to the same control as electronic records?	☐ Yes ☐ No	N/A
11.50 (b)	Are all components of the electronic signature (name, date/time of execution, and meaning) included in all human readable form of the electronic record (e.g. display or print out)?	☐ Yes ☐ No	N/A
11.70	Are electronic signatures, or handwritten signatures applied to electronic records, linked to their respective electronic records to ensure that they cannot be excised, copied, or otherwise transferred to falsify an electronic record by ordinary means?	☐ Yes ☒ No	

CV, curriculum vitae; e.g., for example; GeDI, Genotype–phenotype Data Integration Platform; IT, information technology; NTP, Network Time Protocol; N/A, not applicable; SDLC, Software Development Life Cycle; SOP, Standard Operating Procedure; UBC, United BioSource LLC.

## Abbreviations

ADR: Adverse drug reaction
AE: Adverse event
A&E: Accident and emergency
BMD: Bone mineral density
CFR: Code of federal regulations
CRF: Case report form
CV: Curriculum vitae
DARWIN: Data analysis and real world interrogation network
DMP: Data management plan
DOB: Date of birth
DXA: Dual-energy x-ray absorptiometry
e.g.: For example
EMA: European Medicines Agency
EU: European
EUnetHTA: European network for health technology assessment
F: Female
FAIR: Findable, accessible, interoperable, and reusable
FDA: Food and Drug Administration
GeDI: Genotype-phenotype data integration platform
GCP: Good Clinical Practice
HTA: Health Technology Assessment
HRQOL: Health related quality of life
HRU: Health resource utilisation
IOR: IRCCS istituto ortopedico rizzoli
IT: Information technology
ITU: Intensive therapy unit
KOL: Key opinion leader
M: Male
MAH: Marketing authorisation holder
MeSH: Medical subject headings
NTP: Network time protocol
N/A: Not applicable
OI: Osteogenesis imperfecta
PAES: Post authorisation effectiveness study
PASS: Post authorisation safety study
PRO: Patient-reported outcome
SDLC: Software development life cycle
SOP: Standard operating procedure
SRT: SATURN research team
QoL: Quality of life
RD: Rare disease
REQueST: Registry evaluation of quality standards tool
ROI: Registry of osteogenesis imperfecta
RWE: Real-world evidence
RWD: Real-world data
SATURN: Systematic accumulation of treatment practices and utilisation, real world evidence, and natural history data
TCAE: Terminology criteria for adverse events
US: United States
VAS: Visual analogue scale

## Footnotes

1 The SATURN Research Team includes a group of epidemiologists, statisticians, data management specialists, a project manager and a representative from the Sponsor (Mereo BioPharma).

## References

[1] Arlett P, Kjaer J, Broich K, Cooke E. Real-world evidence in EU medicines regulation: enabling use and establishing value. Clin Pharmacol Ther. 2022;111(1):21–3. 10.1002/cpt.2479. 34797920 10.1002/cpt.2479 PMC9299492

[2] DARWIN EU^®^. Data Analysis and Real World Interrogation Network. https://www.darwin-eu.org/. Accessed: 2 August 2024.

[3] EUnetHTA (2019). REQueST Tool and its vision paper. https://www.eunethta.eu/request-tool-and-its-vision-paper/. Accessed: 2 August 2024.

[4] EUnetHTA (2022). Individual Practical Guideline Document D4.6 Validity of clinical studies. https://www.eunethta.eu/wp-content/uploads/2022/12/EUnetHTA-21-D4.6-Practical-Guideline-on-validity-of-clinical-studies-v1.0-1.pdf. Accessed: 2 August 2024.

[5] European Commission, Health and Consumers Directorate-General (2011). EudraLex: The Rules Governing Medicinal Products in the European Union. Volume 4. Good Manufacturing Practice Medicinal Products for Human and Veterinary Use – Annex 11: Computerised Systems. https://health.ec.europa.eu/system/files/2016-11/annex11_01-2011_en_0.pdf. Accessed: 31 July 2024.

[6] FDA (2003). Part 11, Electronic Records; Electronic Signatures – Scope and Application – Guidance for Industry. https://www.fda.gov/regulatory-information/search-fda-guidance-documents/part-11-electronic-records-electronic-signatures-scope-and-application. Accessed: 31 July 2024.

[7] FDA (2023). Real-world evidence. https://www.fda.gov/science-research/science-and-research-special-topics/real-world-evidence. Accessed: 2 August 2024.

[8] Jansen P, Berg LVD, Overveld PV, Boiten JW. Research Data Stewardship for healthcare professionals. In: Fundamentals of Clinical Data Science [Internet]. Cham (CH): Springer; 2019. Chapter 4. 2018 Dec 22. https://www.ncbi.nlm.nih.gov/books/NBK543528/. Accessed: 2 August 2024. 31314246

[9] Mordenti M, Boarini M, Banchelli F, Antonioli D, Corsini S, Gnoli M, Locatelli M, Pedrini E, Staals E, Triosolino LM, Sangiorgi L. Osteogenesis imperfecta: a cross-sectional study of skeletal and extraskeletal features in a large cohort of Italian patients. Front Endocrinol. 2024;14:1299232. 10.3389/fendo.2023.1299232. 10.3389/fendo.2023.1299232 PMC10809148 38274230

[10] Sangiorgi L, Boarini M, Westerheim I, Skarberg RT, Clancy J, Wang V, Mordenti M. Project SATURN – a real-world evidence data collaboration with existing European datasets in osteogenesis imperfecta to support future therapies. Orphanet J Rare Dis. 2024;19(1):184. 10.1186/s13023-024-03185-y. 38698457 10.1186/s13023-024-03185-y PMC11064334

[11] Wilkinson MD, Dumontier M, Aalbersberg IJ, et al. The FAIR guiding principles for scientific data management and stewardship. Sci Data. 2016;15(3): 160018. 10.1038/sdata.2016.18. 10.1038/sdata.2016.18 PMC4792175 26978244

